# The Role of Brachytherapy in the Management of Oral Squamous Cell Carcinoma: A Systematic Review

**DOI:** 10.3390/jcm14176033

**Published:** 2025-08-26

**Authors:** Fariba Esperouz, Mauro Lorusso, Alfredo De Lillo, Khrystyna Zhurakivska, Lorenzo Lo Muzio, Domenico Ciavarella, Lucio Lo Russo

**Affiliations:** Department of Clinical and Experimental Medicine, School of Dentistry, University of Foggia, 71122 Foggia, Italy; mauro.lorusso@unifg.it (M.L.); alfredo.delillo@unifg.it (A.D.L.); khrystyna.zhurakivska@unifg.it (K.Z.); domenico.ciavarella@unifg.it (D.C.); lucio.lorusso@unifg.it (L.L.R.)

**Keywords:** brachytherapy, carcinoma, squamous cell, high-dose-rate brachytherapy, low-dose-rate brachytherapy, mouth neoplasms

## Abstract

**Background:** This systematic review evaluates the effectiveness and safety of brachytherapy (BT), including low-dose-rate (LDR) and high-dose-rate (HDR) techniques, in the treatment of oral squamous cell carcinoma (OSCC). **Methods:** A systematic search was conducted in PubMed, Scopus, and Web of Science up to April 2025, according to PRISMA guidelines. The review was registered in PROSPERO (CRD42024581512). Eligible studies included cohort, case-control, and longitudinal studies in English investigating BT in OSCC patients. Risk of bias was assessed using ROBINS-I. **Results:** A total of 26 studies with 2286 patients were included where BT was employed as primary or adjuvant therapy, primarily for tumors of the tongue and floor of the mouth. Local control rates ranged from 72% to 95% for both LDR and HDR. HDR BT showed similar efficacy to LDR and offered logistical advantages. Acute and late toxicities included mucositis, soft tissue necrosis, and osteoradionecrosis, particularly with higher doses and large volumes. Combined BT and external beam radiotherapy (EBRT) improved outcomes in selected patients. **Conclusions:** BT remains an effective, organ-preserving option for early-stage OSCC. HDR BT is increasingly adopted due to its comparable efficacy and improved practicality. Optimal patient selection and precise dosimetric planning are crucial to minimize complications. Further prospective studies are warranted to define its role in modern multimodal treatment strategies.

## 1. Introduction

Oral squamous cell carcinoma (OSCC) is a major public health challenge, with an estimated 389,000 new cases diagnosed globally each year [1]. It constitutes the predominant subtype of head and neck cancers, making up roughly 90% of all oral neoplasms. Although the oral cavity is readily accessible for clinical inspection, OSCC is frequently identified at advanced stages, and its incidence continues to rise [2].

The treatment of OSCC depends on the stage; in the early stage the use of chemotherapy (CT) and surgery approach is common; in the advanced stage it is common to combine surgery, radiotherapy, and CT [3,4].

Conventional radiotherapy, or external beam radiation therapy (EBRT), distributes high-energy radiation from an external source, permitting the treatment of larger tumor volumes (>2 cm), oral commissure, and regional lymph nodes [5]. However, its main limitation is the collateral damage to surrounding healthy tissues, leading to side effects such as mucositis, xerostomia, dysphagia, and, in severe cases, osteoradionecrosis [6].

Despite advancements such as intensity-modulated radiotherapy and volumetric-modulated arc therapy, which improve the dose conformity and reduce toxicity toxicity [7], EBRT still exposes non-cancerous structures to radiation [8].

These limitations highlight the necessity of developing new therapeutic strategies, with the view to minimizing patient discomfort and reducing treatment-related morbidity. Consequently, alternative approaches are being investigated, such as brachytherapy [9]. Compared to external beam radiotherapy, the main advantages of interstitial brachytherapy, where radiation sources are placed directly in the tumor, include a higher dose around the target volume and a shorter overall treatment time [10]. This approach minimizes exposure to adjacent normal tissues, reducing systemic side effects and allowing for dose escalation with improved tumor control [11]. It can be applied as a definitive treatment for early OSCC [12]; as a complementary treatment in combination with surgery; as a local “boost” combined with EBRT to enhance the local dose to the immediate tumor region; or as an option for small-burden persistent or recurrent disease [3].

Brachytherapy can be administered through various techniques depending on the tumor’s location, size, depth of invasion, and clinical indications [13]. The two principal approaches are interstitial brachytherapy and intracavitary brachytherapy, both of which can be used with a low dose-rate or a high dose-rate. Interstitial brachytherapy represents the treatment of choice for early stages of small tumors, regionally localized in the oral cavity or in combination with EBRT, to increase radiation dosage selectively in the tumor region. A detailed description of the technical aspects and procedural differences between LDR and HDR techniques is provided in Supplementary S1.

The aim of this systematic review is to explore the current clinical applications of brachytherapy in patients with OSCC, analyzing oncologic outcomes such as local control and survival, as well as treatment-related toxicity. The review provides a narrative synthesis of the evidence from observational studies, with no formal comparison to alternative therapies.

## 2. Materials and Methods

The current systematic review was performed according to the Preferred Reporting Items for Systematic Reviews and Meta-analyses (PRISMA) standards. The protocol was registered in the PROSPERO database: CRD42024581512.

### 2.1. Search Strategy and Database Screening

An electronic search of the English language literature was conducted up to April 2025 using the following databases: MEDLINE (accessed via PubMed), SCOPUS, and Web of Science.

Furthermore, a direct search was conducted in the bibliographies of all reviewed articles.

The research process was carried out via a combination of mesh terms and free text words, combined using some Boolean operators (AND, OR). The following protocol was used for PUBMED: ((“mouth neoplasm *” [MeSH] OR “oral squamous cell carcinoma”) AND (Brachytherapy)) from 2000 until 2025. Full search strategies for Scopus and Web of Science are provided in the Supplementary Materials (Table S1).

### 2.2. Eligibility Criteria

A publication date restriction was applied, and only studies published between January 2000, and April 2025 were used. Studies were screened based on the following inclusion criteria: (1) English language studies; (2) cohort, case-control, observational retrospective or longitudinal studies; (3) Studies focusing on the role of brachytherapy in the management of OSCC; (4) and studies performed on humans. The exclusion criteria were as follows: (1) Systematic review and meta-analysis; (2) Studies not in English.

### 2.3. Focused PIO Question and Effect Measure

- (P) Participants: Patients with a histological diagnosis of oral squamous cell carcinoma (OSCC).
- (I) Intervention: Brachytherapy (LDR or HDR), either as definitive, adjuvant, or boost treatment.
- (O) Outcome: Oncologic outcomes (local control, overall survival), functional outcomes, and treatment-related toxicity.

### 2.4. Studies Screening and Inclusion

Two authors (FE and ML) independently screened for the retrieved citations, by reading the title and abstract. After considering inclusion and exclusion criteria, the authors came up with a list to screen for full-text eligibility evaluation. The third author (LLR) was involved in making a final decision in case of disagreements.

### 2.5. Data Extraction

Similarly, data extraction was undertaken by two reviewers (FE and ML) independently and results were compared and merged by using an ad hoc extraction sheet. In the event of discrepancy, articles were re-screened in a joint meeting with the third reviewer (LLR). The extraction sheet in Excel format included the following fields: author names, country, number of patients, lesion type, site, stage, type of therapy, source, and dose (Table 1).

### 2.6. Assessment of Risk of Bias

The quality assessment and the risk of bias of the included studies were performed following the criteria of the Newcastle–Ottawa Scale (NOS) a validated tool for evaluating the quality of non-randomized studies, particularly cohort and case-control designs. The NOS uses a star-based system to assess three broad domains: selection of participants, comparability of study groups, and ascertainment of outcomes (for cohort studies) or exposures (for case-control studies). Two reviewers (FE and ML) independently rated each study, and disagreements were resolved through discussion with a third reviewer (LLR). Studies were judged to be of low, moderate, or high risk of bias based on their cumulative NOS scores. Two authors (FE and ML) independently performed such assessments and discrepancies were resolved in a joint meeting with the third reviewer (LLR).

### 2.7. Confidence in Cumulative Evidence

The GRADE methodology will be utilized in order to assess the certainty of the evidence [40]. According to the GRADE evidence profile, the certainty of evidence may be reduced if risk of bias, imprecision (small sample populations), inconsistency (heterogeneity), indirectness (including surgical types), or publication bias is found to exist.

## 3. Results

### 3.1. Study Selection

**Figure 1:**
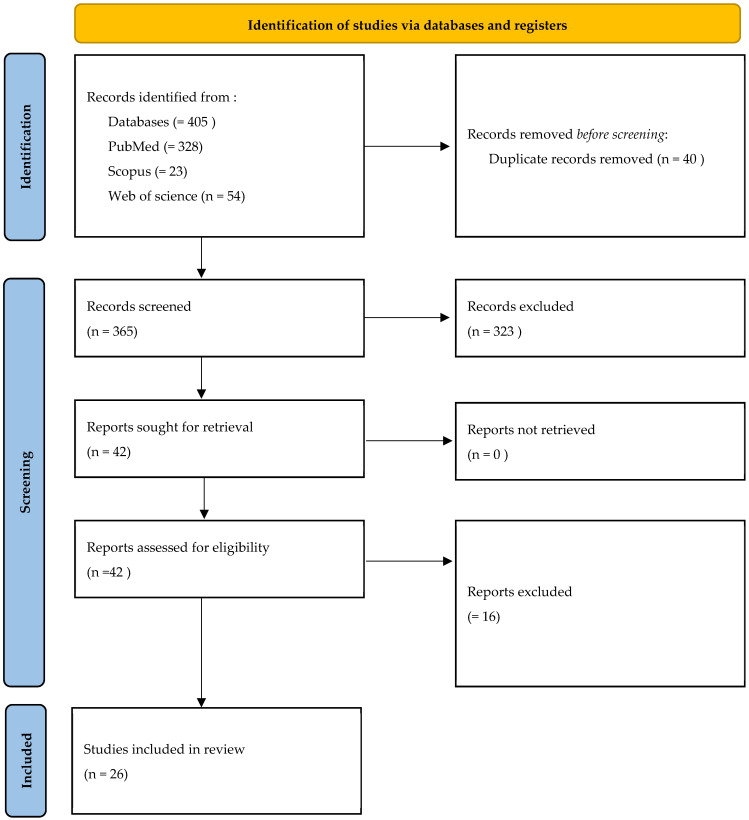
Flowchart of studies’ selection process.

The electronic search yielded a comprehensive compilation of 405 articles, conducted up to June 2025. Following the removal of duplicate entries (40), a meticulous analysis of titles and abstracts was conducted on 365 articles. This process resulted in the exclusion of 323 articles based on initial screening criteria. Subsequently, full-text analysis was applied to the remaining 42 articles, leading to the further exclusion of 16 articles (a detailed list of excluded studies with specific reasons for exclusion is provided in Supplementary Table S2. The final review encompassed a total of 26 articles [15,20,22,39,41]. (Figure 1).

### 3.2. Risk of Bias Assessment

**Figure 2:**
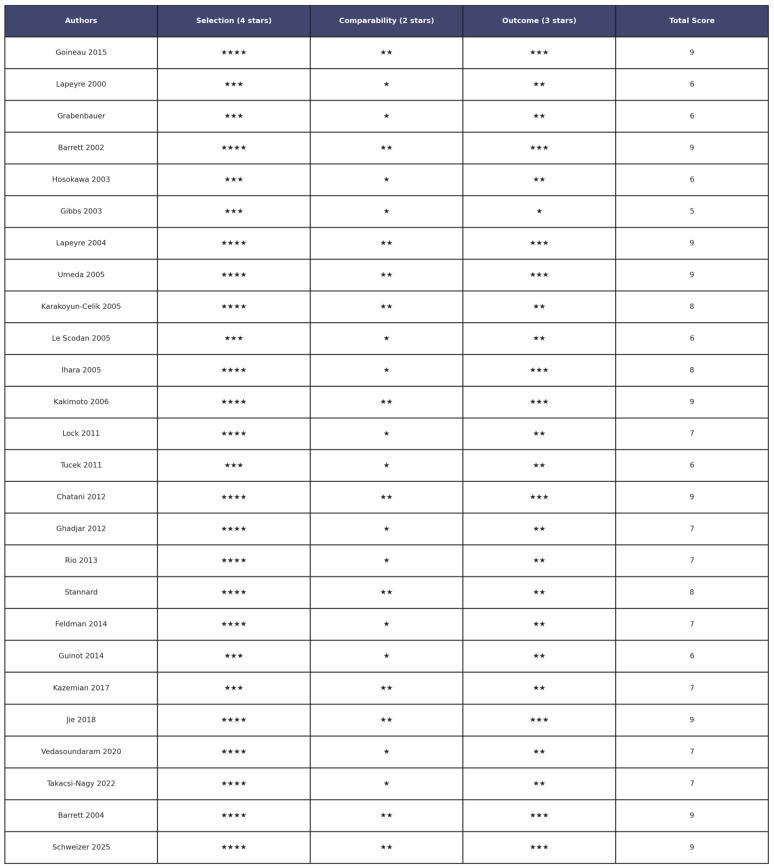
Risk of bias assessment of the 26 articles included in the review articles [15,20,22,39,41] Stars indicate the score assigned for each domain: * = 1 point; ** = 2 points; *** = 3 points; **** = 4 points.

The methodological quality of the 26 included studies [15,20,22,39,41]. was assessed using the Newcastle–Ottawa Scale (NOS) for cohort studies. This tool evaluates three domains: selection (4 points), comparability (2 points), and outcome (3 points), for a maximum total score of 9. Across the dataset, total NOS scores ranged from 5 to 9, reflecting moderate to variable methodological quality. Nine studies [16,19,21,25,28,34,36,39] achieved the highest score of 9, suggesting strong cohort selection, appropriate outcome assessment, and adequate follow-up. Several studies [22,24,31] scored 8 points, reflecting good methodological conduct but with minor limitations. Most other studies scored between 6 and 7 points, showing some weaknesses, often in the comparability domain [3,22,24].In contrast, the study of Gibbs et al. [18] scored 5 points, mainly due to poor reporting or absence of control for confounders, inadequate demonstration of cohort comparability, or insufficient follow-up details. The most frequent sources of bias were the lack of adjustment for key clinical variables (tumor stage, adjuvant treatments, comorbidities), non-blinded outcome assessment, and unclear follow-up duration, which may compromise long-term oncological endpoints. A detailed breakdown of domain-level NOS scores is presented in Supplementary Table S3, while a graphical summary of the distribution is shown in Figure 2. These results highlight the variability in methodological quality, reinforcing the need for caution when interpreting findings, especially where potential confounding and incomplete outcome reporting are present.

### 3.3. Certanty of Evidence

The GRADE approach was used to assess the certainty of evidence across outcomes. Although the included studies were all observational, GRADE can be applied to such designs, starting from a “low” level of certainty by default. Further downgrades or occasional upgrades were made based on five domains: risk of bias, inconsistency, indirectness, imprecision, and publication bias.

For local control, the certainty of evidence was judged as low due to serious risk of bias (non-randomized designs and lack of confounder adjustment) and imprecision (small sample sizes and wide confidence intervals). The certainty for overall survival was also rated as low, downgraded for risk of bias and inconsistency across studies. In contrast, the certainty of evidence for toxicity outcomes was considered very low, reflecting serious methodological concerns and very serious imprecision in adverse event reporting. For disease-free survival, the evidence was rated as very low due to risk of bias, inconsistency, and suspected publication bias (Supplementary Table S4).

### 3.4. Study Characteristics

A total of 26 studies published between 2000 and 2025 were included (Table 1), enrolling 2286 patients with oral squamous cell carcinoma (OSCC). The studies were conducted across multiple countries, including Australia [18], China [36], Czech Republic [27], France (5) [20,23,30,34], Germany (2) [15,39], Hungary [38], India [37], Iran [35], Israel [32]; Japan (5) [17,24,25,28,41], South Africa [31], Spain [33], Switzerland [29], Turkey [22], UK [26], and the USA [16,19].

#### 3.4.1. Population and Tumor Characteristics

Lesion sites were most frequently located on the tongue (*n* = 1294), followed by the floor of the mouth (*n* = 319), lips (*n* = 373), gingiva (*n* = 21), tonsil (*n* = 44), and soft palate (*n* = 60).

Staging information was available for all studies: 24 studies used the 8th edition of the AJCC TNM classification [16,20,22,39], while 2 studies used the UICC staging system studies [15,41]

#### 3.4.2. Brachytherapy Techniques

Low-dose-rate (LDR) interstitial BT was employed in 12 studies [15,20,22,24,26,29,31,34,36,41]. High-dose-rate (HDR) interstitial BT was used in 9 studies [25,27,28,32,33,35,38,39,41], while 2 studies combined HDR with EBRT [35,37]. One study reported using both LDR and HDR techniques in different subgroups [29].The most common isotope was Iridium-192 (Ir-192) (used in 20 studies [15,16,18,20,22,23,25,27,29,30,32,35,37,39,41]), followed by Cesium-137 (2 studies [17,41]), Gold-198 (2 studies [22,26]), Iodine-125 (2 studies [31,36]), and a single study [41] used Radium-226 or mixed sources.

#### 3.4.3. Treatment Setting (Local Control, Overall Survival, and Toxicity)

BT was applied as the definitive treatment for early-stage OSCC (mainly tongue and floor of mouth) and adjuvant therapy or boost after surgery or EBRT for advanced or residual disease.

Outcomes reporting:

- Local control: 72–95% across studies;
- Overall survival: 3- to 5-year OS ranged from 60 to 85%;
- Toxicity: Acute mucositis was the most frequent complication (30–80%). Late complications included soft tissue necrosis (5–20%) and osteoradionecrosis (3–15%).

## 4. Discussion

This systematic review synthesized data from twenty-six observational studies evaluating the clinical role of brachytherapy in the treatment of OSCC, focusing on oncological outcomes.

Overall the findings indicate that interstitial LDR and HDR may offer favorable outcomes in selected patients, especially with early-stage and localized tumors. Across various studies, BT alone or in combination with external beam radiotherapy has consistently shown excellent rates of local tumor control, functional preservation and acceptable toxicity profile. Lapeyre et al. [20] demonstrated that post-operative BT alone for T1-T2 N0 OSCC with close or positive surgical margins achieved excellent local control rates. Grabenbauer et al. [15] confirmed the efficacy of combining post-operative interstitial BT with EBRT.

Goineau et al. [34] showed a 5-year overall survival of 56% and local control of 76% with salvage surgery effective in recurrent cases.

Studies [20,26,27,29,31,33,34] using interstitial LDR BT in early-stage tongue or floor-of-mouth lesions consistently reported high local control rates (>85%) with limited grade 3 toxicities, albeit the cost of prolonged hospitalization.

These results are in line with prior reviews. A work by Draghini et al. [42] focused specifically on LDR BT in tongue carcinoma and concluded that it remains an effective organ-preserving option, with 5-year local control rates around 80–90%. Similarly, the systematic review by Abdulkadir et al. [43] examined exclusively HDR BT in early OSCC, reporting comparable control rates with favorable toxicity profiles.

HDR BT, particularly when image-guided and fractionated appropriately, achieved comparable outcomes with greater convenience and fewer complications. In contrast, BT used for recurrent or advanced OSCC was associated with increased toxicity and reduced survival, especially when administered after multiple prior tratments.

Few studies [21,29] directly compared BT to surgery or ERBT. However, available evidence suggests that for selected T1–T2 OSCC, BT offers comparable oncologic control to surgery or EBRT, with better functional preservation, especially in tongue and lip cancer [15,21,24,29,35]. Recent innovations may expand BT applicability and improve clinical outcomes. Emerging technologies—such as 3D-printed applicators, AI-assisted planning, and robotic catheter insertion—have demonstrated promising results in other oncologic settings [44,45]. These advancements allow for improved dose conformity, reduced procedural time, and enhanced reproducibility, all of which are critical in the anatomically complex head and neck region [46]. Although these technologies have not yet been widely adopted in OSCC, their integration could address some of the logistical and dosimetric limitations historically associated with BT.

The underutilization of BT despite its efficacy may also reflect guideline priorities. Current recommendations from ESTRO and NCCN acknowledge BT as a valid treatment option in early-stage OSCC, particularly for the tongue and lip. However, they generally prioritize surgery or EBRT, often due to limited availability of BT expertise, equipment, and standardization. The incorporation of advanced planning tools and personalized applicators could help reframe BT as a more accessible and standardized treatment modality.

A limited number of studies in this review directly compared brachytherapy (BT) to other established treatment modalities such as surgery or external beam radiation therapy (EBRT) [41]. Nonetheless, emerging evidence suggests that, in selected early-stage OSCC (T1–T2, N0), interstitial BT may offer comparable oncologic outcomes to surgery or EBRT, with the added benefit of preserving function and aesthetics, particularly in anatomically sensitive sites such as the tongue, floor of mouth, and lip [29]. For example, Ghadjar et al. and Umeda et al. reported equivalent local control rates between BT and surgery, but noted superior postoperative function and speech preservation in BT-treated patients.

Despite these promising findings, the lack of randomized controlled trials and direct comparative studies limits the strength of these conclusions. Most evidence derives from small retrospective cohorts, often with heterogeneous populations and inconsistent reporting of outcomes or adverse effects. This heterogeneity precludes definitive recommendations and highlights the need for well-designed prospective studies comparing BT with standard surgical or EBRT-based approaches.

Moreover, current guidelines, including those from ESTRO and NCCN, recognize BT as a treatment option for early-stage OSCC but do not routinely recommend it as a first-line therapy. This may reflect technical and logistical challenges, including the requirement for specialized expertise and limited availability of BT infrastructure.

To improve the comparative positioning of BT, future research should focus on standardizing treatment protocols, incorporating modern technologies (e.g., image guidance, 3D-printed applicators, AI-assisted planning), and evaluating patient-reported outcomes alongside traditional survival metrics. These steps would not only clarify the relative benefits of BT, but also help define its role in the evolving treatment landscape of OSCC.

Future directions include conducting high-quality comparative studies or randomized trials evaluating BT versus surgery or EBRT in OSCC. Additionally, the evaluation of AI-driven planning and personalized dosimetry could be explored in the OSCC setting to reduce toxicity and expand the indications of BT.

## 5. Limitations

This systematic review presents several limitations.

First, all included studies were observational in design—mostly retrospective cohorts or case series—with no randomized controlled trials available. This inherently limits the strength of the evidence and prevents causal inferences.

Second, there was marked heterogeneity across studies in terms of patient populations, tumor sites and stages, brachytherapy techniques (LDR vs. HDR), radiation doses, treatment combinations (definitive, adjuvant, or boost), and outcome measures. Such variability precluded quantitative synthesis and subgroup analyses.

Third, outcome reporting was often incomplete or inconsistent: while some studies reported detailed local control, survival, and toxicity data, others only provided partial or descriptive findings.

Fourth, the possibility of publication bias cannot be excluded, as positive studies are more likely to be published, and most studies had small sample sizes.

Finally, although the risk of bias was assessed using the NOS, most studies received only moderate scores, mainly due to issues in comparability, follow-up duration, or outcome ascertainment, reflecting the methodological limitations of the evidence base.

## 6. Conclusions

Brachytherapy remains a valid treatment option for selected cases of early-stage OSCC, offering good local control and functional preservation. However, the evidence is limited by heterogeneity, outdated protocols, and the lack of high-quality comparative studies. Further prospective research is needed to clarify its role in the era of modern radiotherapy, integrating standardized outcomes and exploring new technologies such as AI-guided planning and robotic-assisted delivery.

## Figures and Tables

**Table 1:** Characteristics of included studies.

Author	Country	Study Design	N° Patients	Lesion Type	Site	Stage	Therapy	Source	Dose	Local Control	Overall Survival (5 Years)	Toxicity
Lapayre 2000 [14]	France	Retrospective study	36	OSCC	Tongue (19) Floor of the mouth (17)	T1(24) T2 (12)	LDR-BT	Ir 192	60 Gy	88.5%	85%	Necrosis
Grabenbauer 2001 [15]	Germany	Retrospective study	318	OSCC	Floor of the mouth (99) Tongue (169) Lower lip (19) Tonsil (19) Others (12)	UICC I (61) II (71) III (58) IV (128)	LDR-BT	Ir 192	45 Gy	74%	54%	Necrosis and osteonecrosis
Barrett 2002 [16]	USA	Retrospective study	20	OSCC	Tongue	T1N2 (1) T1N3 (1) T2 N0 (2) T2N1 (1) T2N2 (7) T2N3 (1) T3N2 (3) T3N3 (1) T4N3 (3)	BT + EBRT	Ir 192	40 Gy	86%	30%	NR
Hosokawa 2003 [17]	Japan	Retrospective study	94	OSCC	Tongue	T1 (29) T2 (65)	LDR-BT + EBRT	Cs 137	40Gy	92.8%	78.4%	Ulcer
Gibbs 2003 [18]	Australia	Prospective study	41	OSCC	Tongue	T1N0 (1) T1N2 (5) T1N3 (1) T2N0 (6) T2N1 (3) T2N2 (4) T2N3 (1) T3N0 (2) T3N1 (2) T3N2 (6) T4N0 (4) T4N2 (4) T4N3 (2)	LDR-BT + EBRT	Ir 192	26 Gy	82%	66%	Mucositis Ulcer
Barrett 2004 [19]	USA	Retrospective study	20	OSCC	Tongue	T1N2 (1) T1N3 (1) T2N0 (2) T2N1 (1) T2N2 (7) T2N3 (1) T3N2 (3) T2N3 (1) T4N3 (3)	LDR-BT + EBRT	Ir 192	25 Gy	89%	87%	NR
Lapeyre 2004 [20]	France	Retrospective study	82	OSCC	Tongue (37) Floor of the mouth (45)	T1N0 (28) T1N1 (1) T2N0 (23) T2N1 (5) T2N2 (1) T3N0 (5) T3N1 (3) T3N2 (2) T4N0 (7) T4N1 (4) T4N2 (2) T4N3 (1)	LDR-BT + EBRT	Ir 192	60 Gy	67%	44%	Necrosis
Umeda 2000 [21]	Japan	Retrospective study	175	OSCC	Tongue	I (92) II (83)	LDR-BT, HDR-BT	Rd 226 Cs 137 Ir 192	61 Gy 59 Gy	91% (LDR) 85% (HDR)	84.0% (LDR) 72.9% (HDR)	Ulcer osteonecrosis
Karakoyun-Celik 2005 [22]	Turkey	Cohort study	122	OSCC	Tongue (104) Floor of the mouth (10) Tonsil (5) Other (3)	T1 (8) T2 (11) T3 (16) T4 (5)	LDR-BT	Ir 192 Au 198	80 Gy	78%	62%	Trismus Swallowing dysfunction Chronic throat pain Osteonecrosis
Le Scodan 2005 [23]	France	Retrospective study	44	OSCC	Tonsil (10) Soft palate (8) Tonsil soft palate (26)	T1N0 (29) T1N1 (1) T1N2 (1) T2N0 (12) T2N1 (1)	LDR-BT	Ir 192	66 Gy	98%	76%	Necrosis
Ihara 2005 [24]	Japan	Retrospective study	117	OSCC	Tongue	T1N1 (1) T2N1 (28) T3N0 (68) T3N1 (20)	LDR-BT	Au 198/Rn-222 (21) Cs-137/Ra-226 (63) Ir 192 (33)	70 Gy	NR	50%	Mucositis
Kakimoto 2006 [25]	Japan	Randomized controlled trial	71	OSCC	Tongue	T1N0 (28) T2N0 (43)	HDR-BT	NR	57 Gy	94%	81%	Necrosis
Lock 2011 [26]	UK	Retrospective study	51	OSCC	Lip	T1 (46) T2 (5)	LDR-BT	Au 198	55 Gy	97.9%	87.9%	Swelling and dry skin
Tuček 2011 [27]	Czech Republic	Retrospective study	20	OSCC	Tongue	T1 (16) T2 (4)	HDR-BT	Ir 192	3 Gy twice a day	85%	75%	Necrosis and osteonecrosis
Chatani 2012 [28]	Japan	Retrospective study	9	OSCC	Oral floor (6) Gingiva (2) Soft palate (1)	T1N0 (7) T2N0 (2)	HDR-BT	GBq 370 Ir 192	9-24 Gy	100%	100%	Ulcer
Ghadjar 2012 [29]	Switzerland	Retrospective study	103	OSCC	Lip	T1 (20; 43) T2 (9; 25) T3 (3; 9)	LDR-BT + HDR-BT	Ir 192	60 Gy LDR 3.5 Gy HDR	93%	77%	Dermatitis (49%), pain (42%), mucositis (41%).
Rio 2013 [30]	France	Retrospective study	89	OSCC	Lip	T1 (44) T2 (33) T3 (2)	LDR-BT	Ir 192	58 Gy	95%	82%	Desquamation
Stannard 2014 [31]	Africa	Retrospective study	112	OSCC	Tongue (54) Soft palate (25) Floor of the mouth (21) Tonsil (10)	T1 (46) T2 (56) T3 (10)	LDR-BT	I 125	0.5 Gy	80.7%	74.3%	Ulcer
Feldman 2014 [32]	Israel	Retrospective study	7	OSCC	Lip	T2 (7)	HDR-BT	Ir 192	25-42 Gy	90%	NR	Mucositis and lip edema
Guinot 2014 [33]	Spain	Retrospective study	102	OSCC	Lip	T1 (54) T2 (33) T4 (15)	HDR-BT	Ir 192	4.5 Gy	95%	85%	Telan- giectasia
Goineau 2015 [34]	France	Retrospective study	112	OSCC	Tongue	T1N0(55) T2N0 (40) T1N1 (1) T2N1 (10) T2N2 (5)	LDR-BT	Ir 192	50-55 Gy	79%	72%	Necrosis and chronic pain
Kazemian 2017 [35]	Iran	Retrospective study	78	OSCC	Tongue (70) Floor of the mouth (4) Lips (2) Buccal mucosa (2)	T1N0 (42) T1N1 (7) T2N0 (14) T2N1 (8) T3N0 (5) T3N1 (2)	HDR-BT + EBRT	Ir 192	39 Gy (HDR) 15 Gy (HDR+EBRT)	90%	83%	Trismus catheter insertion site fibrosis.
Jie 2018 [36]	China	Retrospective study	76	OSCC	Tongue (27) Gingiva (19) Buccal mucosa (8) Floor of the mouth (8) Others (14)	T1 (27) T2 (24) T3 (25)	LDR-BT	I 125	60-160 Gy	95.3%	81.5%	Necrosis
Vedasoundaram 2020 [37]	India	Retrospective study	125	OSCC	Tongue (75) Buccal mucosa (43) Floor of the mouth (7)	T1 (15) T2 (53) T3 (57)	HDR-BT + EBRT	Ir 192	21 Gy	80%	83%	Mucositis and ulcer
Takácsi-Nagy 2022 [38]	Hungary	Retrospective study	45	OSCC	Tongue	T1 (21) T2 (22) T3 (2)	HDR-BT	Ir 192 GBq 370	29 Gy	85%	73%	Mucositis and ulcer
Schweizer 2025 [39]	Germany	s	217	OSCC	Tongue (115) Floor of the mouth (102)	T1 (72) T2 (107) T3 (34) T4 (4)	HDR-BT	Ir 192	34.2 Gy	89.7%	94%	Necrosis and osteonecrosis

BT = Brachytherapy; LDR-BT = Low-Dose-Rate Brachytherapy (typically 0.4–2 Gy/h); HDR-BT = High-Dose-Rate Brachytherapy (>12 Gy/h); BT + EBRT = Brachytherapy combined with External Beam Radiotherapy; Gy = Gray (unit of absorbed radiation dose); UICC = Union for International Cancer Control (alternative staging system); NR = Not Reported.

## Supplementary Materials

The following supporting information can be downloaded at https://www.mdpi.com/article/10.3390/jcm14176033/s1, Supplementary S1. Technical description of brachytherapy procedures, Supplementary Table S1 (Search Strategy), Supplementary Table S2 (Reasons for Exclusion),Supplementary Table S3 (Risk of Bias) and Supplementary Table S4 (GRADE).

## Abbreviations

The following abbreviations are used in this manuscript:

OSCC	Oral squamous cell carcinoma
CT	Chemotherapy
EBRT	external beam radiation therapy
LDR-BT	Low-dose-rate interstitial brachytherapy
LDR	low dose rate
HDR	high dose rate
Ir 192	Iridium-192
C 137	Caesium-137
I 125	Iodine-125

## References

[1] Bray F., Laversanne M., Sung H., Ferlay J., Siegel R.L., Soerjomataram I., Jemal A. (2024). Global cancer statistics 2022: GLOBOCAN estimates of incidence and mortality worldwide for 36 cancers in 185 countries. CA Cancer J. Clin..

[2] Esperouz F., Ciavarella D., Lorusso M., Santarelli A., Muzio L.L., Campisi G., Russo L.L. (2025). Critical review of OCT in clinical practice for the assessment of oral lesions. Front. Oncol..

[3] Huang S.-H., O’Sullivan B. (2013). Oral cancer: Current role of radiotherapy and chemotherapy. Med. Oral Patol. Oral Y Cir. Bucal..

[4] Esperouz F., Caponio V.C.A., Santarelli A., Ballini A., Muzio L.L., Ciavarella D., Russo L.L. (2024). Are we ready to use ultrasounds in the clinical assessment of depth of invasion and tumor thickness in oral squamous cell carcinoma? Results from a systematic review, meta-analysis and trial sequential analysis. Oral Oncol..

[5] Bhalavat R., Pareek V., Chandra M., Nellore L., George K., Borade D., Kalariya K., Moosa Z., Srivastava A., Reddy N. (2018). High-dose-rate interstitial brachytherapy in recurrent head and neck cancer: An effective salvage option. J. Contemp. Brachyther..

[6] Sankar V., Xu Y. (2023). Oral Complications from Oropharyngeal Cancer Therapy. Cancers.

[7] Hunte S.O., Clark C.H., Zyuzikov N., Nisbet A. (2022). Volumetric modulated arc therapy (VMAT): A review of clinical outcomes-what is the clinical evidence for the most effective implementation?. Br. J. Radiol..

[8] Koka K., Verma A., Dwarakanath B.S., Papineni R.V.L. (2022). Technological Advancements in External Beam Radiation Therapy (EBRT): An Indispensable Tool for Cancer Treatment. Cancer Manag. Res..

[9] Mayer C., Gasalberti D.P., Kumar A. (2025). Brachytherapy. StatPearls.

[10] Vavassori A., Gherardi F., Colangione S.P., Fodor C., Cattani F., Lazzari R., Calabrese L., Bruschini R., Alterio D., Jereczek-Fossa B.A. (2012). High-dose-rate interstitial brachytherapy in early stage buccal mucosa and lip cancer: Report on 12 consecutive patients and review of the literature. Tumori.

[11] Lee C.D. (2014). Recent developments and best practice in brachytherapy treatment planning. Br. J. Radiol..

[12] Mazeron J.J., Ardiet J.M., Haie-Méder C., Kovács G., Levendag P., Peiffert D., Polo A., Rovirosa A., Strnad V. (2009). GEC-ESTRO recommendations for brachytherapy for head and neck squamous cell carcinomas. Radiother. Oncol..

[13] Muto P., Pastore F. (2021). Radiotherapy in the Adjuvant and Advanced Setting of CSCC. Dermatol. Pract. Concept..

[14] Lapeyre M., Hoffstetter S., Peiffert D., Guérif S., Maire F., Dolivet G., Toussaint B., Mundt A., Chassagne J.-F., Simon C. (2000). Postoperative brachytherapy alone for T1-2 N0 squamous cell carcinomas of the oral tongue and floor of mouth with close or positive margins. Int. J. Radiat. Oncol. Biol. Phys..

[15] Grabenbauer G.G., Rödel C., Brunner T., Schulze-Mosgau S., Strnad V., Müller R.G., Iro H., Sauer R. (2001). Interstitial brachytherapy with Ir-192 low-dose-rate in the treatment of primary and recurrent cancer of the oral cavity and oropharynx. Review of 318 patients treated between 1985 and 1997. Strahlenther. Onkol..

[16] Barrett W.L., Gleich L., Wilson K., Gluckman J. (2002). Organ preservation with interstitial radiation for base of tongue cancer. Am. J. Clin. Oncol..

[17] Hosokawa Y., Shirato H., Nishioka T., Tsuchiya K., Chang T.-C., Kagei K., Ohomori K., Obinata K.-I., Kaneko M., Miyasaka K. (2003). Effect of treatment time on outcome of radiotherapy for oral tongue carcinoma. Int. J. Radiat. Oncol. Biol. Phys..

[18] Gibbs I.C., Le Q.T., Shah R.D., Terris D.J., Fee W.E., Goffinet D.R. (2003). Long-term outcomes after external beam irradiation and brachytherapy boost for base-of-tongue cancers. Int. J. Radiat. Oncol. Biol. Phys..

[19] Barrett W.L., Gluckman J.L., Wilson K.M., Gleich L.L. (2004). A comparison of treatments of squamous cell carcinoma of the base of tongue: Surgical resection combined with external radiation therapy, external radiation therapy alone, and external radiation therapy combined with interstitial radiation. Brachytherapy.

[20] Lapeyre M., Bollet M.A., Racadot S., Geoffrois L., Kaminsky M., Hoffstetter S., Dolivet G., Toussaint B., Luporsi E., Peiffert D. (2004). Postoperative brachytherapy alone and combined postoperative radiotherapy and brachytherapy boost for squamous cell carcinoma of the oral cavity, with positive or close margins. Head Neck.

[21] Umeda M., Komatsubara H., Nishimatsu N., Yokoo S., Shibuya Y., Komori T. (2000). High-dose rate interstitial brachytherapy for stage I-II tongue cancer. Oral Surg. Oral Med. Oral Pathol. Oral Radiol..

[22] Karakoyun-Celik O., Norris C.M.J., Tishler R., Mahadevan A., Clark J.R., Goldberg S., Devlin P., Busse P.M. (2005). Definitive radiotherapy with interstitial implant boost for squamous cell carcinoma of the tongue base. Head Neck.

[23] Le Scodan R., Pommier P., Ardiet J.M., Montbarbon X., Malet C., Favrel V., Zrounba P., Poupart M., Céruse P., Ferlay C. (2005). Exclusive brachytherapy for T1 and T2 squamous cell carcinomas of the velotonsillar area: Results in 44 patients. Int. J. Radiat. Oncol. Biol. Phys..

[24] Ihara N., Shibuya H., Yoshimura R., Oota S., Miura M., Watanabe H. (2005). Interstitial brachytherapy and neck dissection for Stage III squamous cell carcinoma of the mobile tongue. Acta Oncol..

[25] Kakimoto N., Inoue T., Inoue T., Murakami S., Furukawa S., Yoshida K., Yoshioka Y., Yamazaki H., Tanaka E., Kimishige Shimizutani K. (2006). High-dose-rate interstitial brachytherapy for mobile tongue cancer: Influence of the non-irradiated period. Anticancer Res..

[26] Lock M., Cao J.Q., D’Souza D.P., Hammond J.A., Karnas S., Lewis C., Venkatesan V.M., Whiston E., Yau G., Yu E. (2011). Brachytherapy with permanent gold grain seeds for squamous cell carcinoma of the lip. Radiother. Oncol..

[27] Tuček L., Petera J., Sirák I., Vošmik M., Doležalová H., Brokešová S., Hodek M., Kašaová L., Paluska P. (2011). Hyperfractionated high-dose rate brachytherapy in the treatment of oral tongue cancer. Rep. Pract. Oncol. Radiother..

[28] Chatani M., Tsuboi K., Yagi M., Fujiwara K., Tachimoto R., Yoshioka H. (2012). High dose rate brachytherapy using molds after chemoradiotherapy for oral cavity cancer. Jpn. J. Radiol..

[29] Ghadjar P., Bojaxhiu B., Simcock M., Vošmik M., Doležalová H., Brokešová S., Hodek M., Kašaová L., Paluska P. (2012). High dose-rate versus low dose-rate brachytherapy for lip cancer. Int. J. Radiat. Oncol. Biol. Phys..

[30] Rio E., Bardet E., Mervoyer A., Piot B., Dreno B., Malard O. (2013). Interstitial brachytherapy for lower lip carcinoma: Global assessment in a retrospective study of 89 cases. Head Neck.

[31] Stannard C., Maree G., Tovey S., Hunter A., Wetter J. (2014). Iodine-125 brachytherapy in the management of squamous cell carcinoma of the oral cavity and oropharynx. Brachytherapy.

[32] Feldman J., Appelbaum L., Sela M., Voskoboinik N., Kadouri S., Weinberger J., Orion I., Meirovitz A. (2014). Novel high dose rate lip brachytherapy technique to improve dose homogeneity and reduce toxicity by customized mold. Radiat. Oncol..

[33] Guinot J.L., Arribas L., Vendrell J.B., Santos M., Tortajada M.I., Mut A., Cruz J., Mengual J.L., Chust M.L. (2014). Prognostic factors in squamous cell lip carcinoma treated with high-dose-rate brachytherapy. Head Neck.

[34] Goineau A., Piot B., Malard O., Ferron C., Lisbona A., Cassagnau E., Delamazure A.-S., Campion L., Bardet E. (2015). Postoperative interstitial brachytherapy for resectable squamous cell carcinoma of the tongue. Brachytherapy.

[35] Kazemian A., Babaei M., Lashkari M., Ghalehtaki R., Garajei A., Motiee-Langroudi M., Sebzari A., Jaberi R., Gholami S., Babaloui S. (2017). Adjuvant high-dose-rate brachytherapy in the management of oral cavity cancers: 5 years of experience in Iran. J. Contemp. Brachyther..

[36] Jie W.P., Bai J.Y., Li B.B. (2018). Clinicopathologic Analysis of Oral Squamous Cell Carcinoma After (125)I Interstitial Brachytherapy. Technol. Cancer Res. Treat..

[37] Vedasoundaram P., Raghava Ks A., Periasamy K., Selvarajan G., K S., Kandasamy S., R S., Kumar A. (2020). The Effect of High Dose Rate Interstitial Implant on Early and Locally Advanced Oral Cavity Cancers: Update and Long-Term Follow-Up Study. Cureus.

[38] Takácsi-Nagy Z., Ferenczi Ö., Major T., Akiyama H., Fröhlich G., Oberna F., Révész M., Poósz M., Polgár C. (2022). Results of sole postoperative interstitial, high-dose-rate brachytherapy of T1-2 tongue tumours. Strahlenther. Onkol..

[39] Schweizer C., Strnad V., Lotter M., Kreppner S., Merten R., Fietkau R., Karius A. (2025). Postoperative brachytherapy alone for 217 patients with early-stage oral cavity squamous cell carcinoma. Clin. Transl. Radiat. Oncol..

[40] Granholm A., Alhazzani W., Møller M.H. (2019). Use of the GRADE approach in systematic reviews and guidelines. Br. J. Anaesth..

[41] Umeda M., Komatsubara H., Ojima Y., Minamikawa T., Shibuya Y., Yokoo S., Ishii J., Komori T. (2005). A comparison of brachytherapy and surgery for the treatment of stage I-II squamous cell carcinoma of the tongue. Int. J. Oral Maxillofac. Surg..

[42] Draghini L., Lancellotta V., Fionda B., De Angeli M., Cornacchione P., Massaccesi M., Trippa F., Kovács G., Morganti A.G., Bussu F. (2024). Can interventional radiotherapy (brachytherapy) be an alternative to surgery in early-stage oral cavity cancer? A systematic review. Strahlenther. Onkol..

[43] Abdulkadir M.K., Appalanaido G.K., Musa M.Y., Bin Jalil J., Bin Mohamad A.F., Yogabalan K., Aziz M.Z.A. (2025). Sole high dose-rate interstitial brachytherapy for early-stage tongue cancer: A systematic review. Cancer Radiother..

[44] Shi J., Chen J., He G., Peng Q. (2025). Artificial intelligence in high-dose-rate brachytherapy treatment planning for cervical cancer: A review. Front. Oncol..

[45] Banerjee S., Goyal S., Mishra S., Gupta D., Bisht S.S., K V., Narang K., Kataria T. (2021). Artificial intelligence in brachytherapy: A summary of recent developments. Br. J. Radiol..

[46] Wong K.C.W., Johnson D., Hui E.P., Lam R.C.T., Ma B.B.Y., Chan A.T.C. (2022). Opportunities and challenges in combining immunotherapy and radiotherapy in head and neck cancers. Cancer Treat. Rev..

