# Supplementary material for: The Role of Brachytherapy in the Management of Oral Squamous Cell Carcinoma: A Systematic Review
Source: J Clin Med. 2025 Aug 26;14(17):6033. doi: 10.3390/jcm14176033 (PMC12429441; doi:10.3390/jcm14176033)
Supplement: Supplementary file 1 [file jcm-14-06033-s001.zip › Supplementary S1.pdf]

**Supplementary S1. Technical description of brachytherapy procedures**

## 1. Interstitial brachytherapy

Interstitial brachytherapy involves the direct implantation of radioactive sources into the tumor or peritumoral tissue using needles, catheters, or flexible plastic tubes {de Souza Lawrence, 2017 #238085}. The implantation process is guided by imaging techniques such as ultrasound, CT, or MRI, ensuring optimal placement of the radiation sources and avoiding blood vessels and other critical structures {Esperouz et al., 2024 #161511; Jacobsen et al., 2022 #113298}.

This treatment approach reduces the overall radiation dose and treatment duration, while minimizing radiotherapy-related complications and patient discomfort by ensuring precise positioning and dose measurement. These advantages contribute to improved clinical outcomes {Tian et al., 2018 #135171}.

## 2. Low-dose-rate (LDR) brachytherapy

LDR brachytherapy delivers a continuous radiation dose at a rate of **0.4–2 Gy/h** through the insertion of plastic catheters or needles. Treatment durations range from **24–144 hours (1–6 days)** {Yavaş, 2019 #225441}. Due to the prolonged exposure time, patients must be hospitalized during treatment, which represents a significant disadvantage.

The most commonly used isotopes include **Iridium-192 (Ir-192)**, **Caesium-137 (Cs-137)**, and **Iodine-125 (I-125)**.

## 3. High-dose-rate (HDR) brachytherapy

In contrast to LDR, HDR brachytherapy delivers radiation in short, high-intensity pulses typically lasting only a few minutes per session {Yavaş, 2019 #225441}. This technique allows outpatient treatment, offering greater scheduling flexibility and eliminating the need for prolonged hospitalization.

HDR is well-suited for modern image-guided planning, allowing precise dose delivery through optimized dwell times. It reduces radiation exposure to healthy tissues and improves safety for staff, as radiation is only delivered during controlled sessions. HDR is also more efficient, requiring a single radiation source and reducing procedural complexity {Choi et al., 2018 #290834; Yamazaki et al., 2013 #90543}.

However, HDR requires careful dosimetric planning, as the higher dose per fraction can increase toxicity risk if inaccurately delivered {Feldman et al., 2014 #204756}. Extending the fraction time to reduce dose rate can also prolong sessions, requiring a balance between effectiveness and patient comfort {Glatzel et al., 2002 #51445}.

**4. Intracavitary brachytherapy**

Intracavitary brachytherapy involves the use of applicators to deliver radioactive sources into natural body cavities (e.g., oral or vaginal cavity) or surgically created spaces (e.g., lumpectomy cavity) {Funk et al., 2016 #137570}.

- **LDR intracavitary brachytherapy:** Continuous delivery of radiation at **0.4–2 Gy/h** for **24–96 hours** requires hospitalization in radiation-protected rooms, which poses logistical challenges despite radiobiological advantages {Mayer et al., 2025 #184735; Scott et al., 2021 #202218}.
- **HDR intracavitary brachytherapy:** Short, high-intensity pulses (lasting a few minutes per fraction) are delivered using high-activity sources such as Iridium-192 {Lim & Kim, 2021 #70342}. Advanced computer-based planning systems optimize dwell times, allowing outpatient treatments with improved safety and patient convenience.

HDR has largely replaced LDR in most modern clinical settings, although LDR remains in use in selected cases. Intracavitary procedures are generally simpler than interstitial techniques and require less procedural expertise but carry a greater risk of operational errors, such as incorrect source strength selection or source misplacement. To reduce these risks, HDR delivery systems with remote afterloading are now widely adopted, improving safety, accuracy, and standardization {Funk et al., 2016 #137570}.
