# Supplementary material for: The Role of Brachytherapy in the Management of Oral Squamous Cell Carcinoma: A Systematic Review
Source: J Clin Med. 2025 Aug 26;14(17):6033. doi: 10.3390/jcm14176033 (PMC12429441; doi:10.3390/jcm14176033)
Supplement: Supplementary file 1 [file jcm-14-06033-s001.zip › Supplementary Table 2.pdf]

Author (Year)	Reason for exclusion	Reference
Agarwala, 2006	Not original research / Not relevant	PMID: 17348432
Yoshida, 2017	Focused only on complications	PMID: 23732769
Leborgne et al., 2002	Wrong intervention (Cs-137 only)	PMID: 15090274
Konishi et al., 2021	Evaluated physicians rather than patients	PMID: 37831428
Ganly et al., 2012	Wrong intervention (surgery only, no BT)	PMID: 23184439
Olmos et al., 2021	Immunotherapy	PMID: 34368002
Yamazaki et al., 2001	Wrong population (pediatric/age mismatch)	PMID: 11240233
Polo, 2009	Review	PMID: 3343172
Huang & O'Sullivan, 2013	Review	PMID: 23385513
Agaku & Akinyele, 2013	Database study, not primary research	PMID: 23021918.
Hirai et al., 2020	Wrong intervention (postoperative EBRT, no BT)	PMID: 32339995
Prisciandaro et al., 2005	Case report (buccal mucosa, HDR stent)	PMID: 15770193
Tian et al., 2018	Wrong intervention (125I seeds, not standard BT)	PMID: 29630112
Uchimoto et al., 2022	Case report (radiation-induced sarcoma)	PMID: 36199998
Yoshida et al., 2000	Wrong population (patients <40 years old)<br>Wrong population (patients <40 years old)	PMID: 10487557.
Yamazaki et al., 2007	Age-related subgroup analysis, not consistent with inclusion criteria	PMID: 17348432.
