# Supplementary material for: The Role of Brachytherapy in the Management of Oral Squamous Cell Carcinoma: A Systematic Review
Source: J Clin Med. 2025 Aug 26;14(17):6033. doi: 10.3390/jcm14176033 (PMC12429441; doi:10.3390/jcm14176033)
Supplement: Supplementary file 1 [file jcm-14-06033-s001.zip › Supplementary Table 4.pdf]

Outcome	Risk of bias	Inconsistency	Indirectness	Imprecision	Publication bias	Quality of evidence
**Local control**	Serious limitations	No serious limitations	No serious limitations	Serious limitations	No serious limitations	⊕⊕◯◯ **Low**
**Overall survival**	Serious limitations	Serious limitations	No serious limitations	Serious limitations	No serious limitations	⊕⊕◯◯ **Low**
**Toxicity**	Serious limitations	No serious limitations	No serious limitations	Very serious limitations	No serious limitations	⊕◯◯◯ **Very low**
**Disease-free survival**	Serious limitations	Serious limitations	No serious limitations	Serious limitations	Possible publication bias	⊕◯◯◯ **Very low**

Table 3 GRADE profile of the effect of brachytherapy on OSCC
